# Exploring cell cycle-mediated regulations of glycolysis in budding yeast

**DOI:** 10.3389/fmicb.2023.1270487

**Published:** 2023-10-11

**Authors:** Yanfei Zhang, Matteo Barberis

**Affiliations:** ^1^Molecular Systems Biology, School of Biosciences, Faculty of Health and Medical Sciences, University of Surrey, Guildford, United Kingdom; ^2^Synthetic Systems Biology and Nuclear Organization, Swammerdam Institute for Life Sciences, University of Amsterdam, Amsterdam, Netherlands; ^3^Centre for Mathematical and Computational Biology, CMCB, University of Surrey, Guildford, United Kingdom

**Keywords:** cell cycle, metabolism, cyclin-dependent kinase, cyclin B/Cdk1, cyclin-dependent kinase inhibitor, Sic1, metabolic enzymes, glycolysis

## Abstract

Coordination of cell cycle with metabolism exists in all cell types that grow by division. It serves to build a new cell, (i) fueling building blocks for the synthesis of proteins, nucleic acids, and membranes, and (ii) producing energy through glycolysis. Cyclin-dependent kinases (Cdks) play an essential role in this coordination, thereby in the regulation of cell division. Cdks are functional homologs across eukaryotes and are the engines that drive cell cycle events and the clocks that time them. Their function is counteracted by stoichiometric inhibitors; specifically, inhibitors of cyclin-cyclin dependent kinase (cyclin/Cdk) complexes allow for their activity at specific times. Here, we provide a new perspective about the yet unknown cell cycle mechanisms impacting on metabolism. We first investigated the effect of the mitotic cyclin/Cdk1 complex Cyclin B/Cdk1—functional homolog in mammalian cells of the budding yeast Clb2/Cdk1—on yeast metabolic enzymes of, or related to, the glycolysis pathway. Six glycolytic enzymes (Glk1, Hxk2, Pgi1, Fba1, Tdh1, and Pgk1) were subjected to *in vitro* Cdk-mediated phosphorylation assays. Glucose-6-phosphate dehydrogenase (Zwf1), the first enzyme in the pentose phosphate pathway that is important for NADPH production, and 6-phospho-fructo-2-kinase (Pfk27), which catalyzes fructose-2,6-bisphosphate synthesis, a key regulator of glycolysis, were also included in the study. We found that, among these metabolic enzymes, Fba1 and Pgk1 may be phosphorylated by Cdk1, in addition to the known Cdk1-mediated phosphorylation of Gph1. We then investigated the possible effect of Sic1, stoichiometric inhibitor of mitotic cyclin/Cdk1 complexes in budding yeast, on the activities of three most relevant glycolytic enzymes: Hxk2, Glk1, and Tdh1. We found that Sic1 may have a negative effect on Hxk2. Altogether, we reveal possible new routes, to be further explored, through which cell cycle may regulate cellular metabolism. Because of the functional homology of cyclin/Cdk complexes and their stoichiometric inhibitors across evolution, our findings may be relevant for the regulation of cell division in eukaryotes.

## 1. Introduction

Building a new cell entails a sequence of processes with specific demands for biosynthetic precursors and Gibbs energy. These place dynamic requirements on metabolism. *Saccharomyces cerevisiae* (*S. cerevisiae*), one of the microorganisms most used in the microbial production of proteins, chemicals, food, and metabolites, is the most intensively studied unicellular eukaryote. More and more shreds of evidence suggest that *S. cerevisiae* metabolism can be regulated by cell cycle progression, as different biosynthetic pathways are preferentially active in different cell cycle phases (Takhaveev et al., 2023; Zhang et al., 2023).

Under chemostat conditions (under continuous, nutrient-limited conditions) more than half of the yeast genes exhibit periodic expression. NAD(P)H and ATP concentrations oscillate with the cell cycle (Tu et al., 2005). Ewald et al. (2016) showed that metabolic fluxes are coordinated with the cell cycle in what may be called their Metabolic Cell Cycle (MCC). The concentrations of hundreds of metabolites were found changing during the cell cycle process. Most of these metabolites were involved in biosynthetic pathways toward precursors of amino acids, cell wall components, and lipids. Extensive cell cycle-dependent changes were also found in the central carbon metabolism: glycolysis, tricarboxylic acid cycle, and the pentose-phosphate pathway (Ewald et al., 2016). These pathways are, indeed, the major routes that provide precursors for the biosynthetic pathways throughout the cell cycle. Cell cycle-dependent changes in central carbon metabolism are controlled by the cyclin-dependent kinase (Cdk1), which is a major regulator of the cell cycle. The neutral trehalase Nth1 can be phosphorylated and thereby activated by the yeast mitotic Clb2/Cdk1 complex (Ewald et al., 2016). This enzyme funnels the storage carbohydrate trehalose into central carbon metabolism. Glycogen phosphorylase (Gph1), which serves the breakdown of glycogen (a branched polysaccharide), can also be phosphorylated and activated by the recombinant human mitotic Cyclin B/Cdk1 complex (Zhao et al., 2016). These findings directly link storage carbohydrate metabolism with cell cycle control.

Storage carbohydrate metabolism is directly connected with glycolysis. Glycolysis is the metabolic pathway that converts glucose to ethanol and CO_2_ in budding yeast. It can provide Gibbs energy (in the form of ATP) under both aerobic and anaerobic conditions. This pathway also provides precursors for the synthesis of glycolipids, glycoproteins, and structural polysaccharides, which are the constituents of the membranes. In *S. cerevisiae*, three enzymes are mostly involved in the capture of glucose as glucose-6-phosphate, i.e., two hexokinases, Hxk1 and Hxk2, and one glucokinase, Glk1 (Walsh et al., 1983). Hxk2 is the predominant enzyme during growth on a fermentation medium using glucose, fructose or mannose as a carbon source (De Winde et al., 1996; Bianconi, 2003). The other two enzymes are mainly used when growing cells on non-fermentable carbon source (Rodeiguez et al., 2001). It has been reported that Hxk2 also plays a role in the cell cycle, with deletion of Hxk2 significantly enhancing yeast longevity (Kaeberlein et al., 2005). Phosphoglucose isomerase (Pgi), is the second glycolytic enzyme that catalyzes the interconversion of glucose-6-phosphate and fructose-6-phosphate (Aguilera and Zimmermann, 1986); fructose-6-phosphate is a precursor for the cell wall component chitin (polysaccharide) and mannoprotein (containing polysaccharide). Mutation in the *PGI1* gene may cause cell cycle arrest (Dickinson, 1991), thus linking Pgi1 with cell cycle regulation.

6-phospho-fructo-2-kinase (Pfk27) is a key glycolytic enzyme (Rider et al., 2004), catalyzing the synthesis of fructose-2,6-bisphosphate from fructose-6-phosphate and ATP. Fructose-2,6-bisphosphate is not an intermediate in a metabolic pathway, but rather a strong positive allosteric effector of the glycolytic enzyme phosphofructokinase (Pfk1 and Pfk2). In human cells, overexpression of PFKFB3 (homolog of the budding yeast Pfk27) on the one hand induces the expression of several cell cycle proteins such as Cdk1 and Cyclin D3, on the other hand decreases the expression of Cdk1’s stoichiometric inhibitor p27^Kip1^—the mammalian homolog of the budding yeast Sic1 (Barberis et al., 2005; Yalcin et al., 2009).

Fructose-1,6-bisphosphate aldolase (Fba1) catalyzes the reversible reaction of fructose-1,6-biophosphate to glyceraldehyde-3-phosphate and dihydroxyacetone phosphate. This enzyme is required for both glycolysis and gluconeogenesis.

Glyceraldehyde-3-phosphate dehydrogenase (Tdh1, also known as G3PDH or GAPDH) is the first enzyme in the lower part of the glycolytic pathway and catalyzes the reaction from glyceraldehyde-3-phosphate plus phosphate and NAD^+^ to 1,3 bis-phosphoglycerate, NADH and H^+^. NADH is a coenzyme shuttling redox equivalents from intermediary metabolism to oxidative phosphorylation of ADP, thereby serving the cell with ATP. The human glyceraldehyde-3-phosphate dehydrogenase may regulate the activity of Cdk1 by direct association to Cyclin B (Carujo et al., 2001; Lincet and Icard, 2015). In budding yeast, Tdh1 may have an additional non-glycolytic function through association with the cell wall (Delgado et al., 2001). Glucose-6-phosphate dehydrogenase (Zwf1, also known as G6PDH) catalyzes the first step of the pentose phosphate pathway, and it is required for generating NADPH, which is a source of redox equivalents in anabolism toward DNA and proteins. The deletion strain *zwf1*Δ does not undergo the metabolic cell cycle (Tu et al., 2007).

As indicated in the examples above, eukaryotic cyclin-dependent kinases (e.g., Cdk1) and their stoichiometric inhibitors (e.g., p27^Kip1^) may regulate and/or be regulated by metabolic enzymes. Cdk1 is the best-studied cyclin-dependent protein kinase and plays an essential role in the regulation of the cell cycle (Morgan, 1997). It alternately associates with G1, S, or G2/M phase cyclins, and its human homologs Cdk1, Cdk2, and Cdk3 can complement its yeast mutants (Ninomiya-Tsuji et al., 1991; Meyerson et al., 1992). The above reviewed indications of interactions of the cell cycle with metabolic enzymes may occur through direct or indirect mechanisms. Here, we provide a new perspective about these yet unknown mechanisms, by examining the possibility that the interactions are direct, i.e., through cyclin/Cdk-mediated phosphorylation of metabolic enzymes. We focused on the M phase cyclin/Cdk1 complex, because central carbon metabolism products reach their peak levels at the end of the cell cycle, in G2/M phase (Campbell et al., 2020). Specifically, we examined whether yeast metabolic enzymes are phosphorylated by the Cyclin B/Cdk1 complex active in G2/M phase in mammalian cells. This complex drives the M phase, thereby cell division, of the mammalian cell cycle and it is homologous to the Clb2/Cdk1 complex of budding yeast. Thus, the first part of this perspective is focused on investigating the effects of the M phase of the cell cycle on metabolism, which we found to be significant.

With respect to the stoichiometric inhibitors of S– and G/M–phase cyclin/Cdk complexes, the budding yeast Sic1 is synthesized at the end of mitosis and blocks the catalytic activity of Clb2/Cdk1. At this cell cycle phase, the cell needs to decide whether it will enter a next round of the cell cycle or stationary phase (G0). This decision has an implication for metabolism. If the decision is to proceed toward a next round of the cell division cycle, the metabolism needs to be promptly activated. Conversely, if the decision is for G0, the metabolism may need to be repressed. Thus, in the second part of this perspective we will therefore further examine whether Sic1 has a direct effect on the enzyme activity of three most relevant enzymes involved in the activation of the glycolytic pathway: Hxk2 (the dominant hexokinase at high glucose), Glk1 (the only glucokinase), and Tdh1. These enzymes of *S. cerevisiae* were purified and changes in their enzymatic activity after administration of Sic1 were measured. We found that Sic1 may inhibit Hxk2, since Hxk2 activity decreased around 11% after Sic1 administration.

Altogether, we tackle a new perspective on the complex interconnection between cell cycle and metabolism, revealing possible new routes through which cell cycle may regulate cellular metabolism.

## 2. Fba1 and Pgk1 may be phosphorylated by Cdk1 *in vitro*

In the first part of the study, we have explored the possibility for specific glycolytic enzymes to be substrate for the Cdk1 activity. The metabolic enzymes were first isolated and purified (see Supplementary Material and Methods and Supplementary Figures 1A–B). Then, *in vitro* phosphorylation assays were performed, incubating the purified metabolic enzymes with the human Cyclin B/Cdk1 complex, which was added in a concentration similar to that reported in literature to test phosphorylation of enzymes of the carbohydrate metabolism (Ewald et al., 2016; Zhao et al., 2016). The assays were analyzed on Phos-tag SDS-PAGE gels.

**FIGURE 1:**
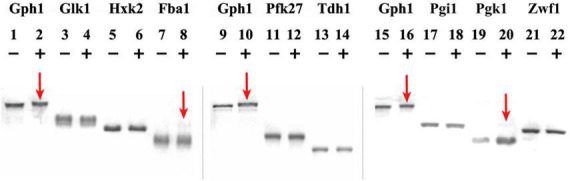
Cyclin B/Cdk1 phosphorylation assay of metabolic enzymes. Gph1, Glk1, Hxk2, Fba1, Pfk27, Tdh1, Pgi1, Pgk1, and Zwf1 were purified from *E. coli* and treated with human Cyclin B/Cdk1 *in vitro*, then analyzed on Phos-tag SDS-PAGE gels. (+) and (−) indicate the presence and absence of Cyclin B/Cdk1 in the assay, respectively. Mn^2+^– Phos-tag™ preferentially captures phospho-monoester dianions (-OPO_3_^2–^) bound to proteins, resulting in a mobility downshift of the phosphorylated proteins on the gel.

Phosphorylation assays are shown in Figure 1, where the migration shift of the bands corresponding to the metabolic enzymes upon phosphorylation reaction with (+) or without (−) Cyclin B/Cdk1 is observed. Gph1 is used as a positive control, as it has been shown to be substrate of Cyclin B/Cdk1 activity (Zhao et al., 2016): lanes 1, 9, 15 and lanes 2, 10, 16 in Figure 1 show the Gph1 band without (−) and with (+) Cyclin B/Cdk1 added to the sample, respectively. It can be observed that the Gph1 band in lanes 2, 10, 16 exhibits a lower mobility (at the higher position of the gel) compared to the same band in lanes 1, 9, 15. Because phosphate affinity SDS-PAGE using Acylamide-pendant Phos-tagTM provides mobility shift detection of phosphorylated proteins, the observed difference between the samples can be attributed to the Cyclin B/Cdk1-mediated phosphorylation of Gph1. For Glk1, Hxk2, Pfk27, Tdh1, Pgi1, and Zwf1, no changes in the mobility of their band are observed on the phosphate affinity SDS-PAGE upon adding Cyclin B/Cdk1 to the samples. The result indicates that these metabolic enzymes are not phosphorylated by Cyclin B/Cdk1.

Conversely, a light band with a higher molecular weight appeared above the main band of Fba1 in the reaction where Cyclin B/Cdk1 was added to the sample (lane 8), indicating that a fraction of Fba1 may have been phosphorylated. The distance between the higher and the main bands is larger than that between the phosphorylated and non-phosphorylated Gph1 bands, suggesting that this higher (light) band represents hyper phosphorylation of Fba1. Similarly to Fba1, a light band with a higher molecular weight appeared above the main band of Pgk1 in the reaction where Cyclin B/Cdk1 was added to the sample (lane 20), indicating that a fraction of Pgk1 may have been phosphorylated.

To further prove the successful phosphorylation essays, a similar Cyclin B/Cdk1 concentration was used to test a well-known positive control, i.e., the phosphorylation of the cell cycle inhibitor Sic1 by Cdk1. This essay clearly shows a complete shift of the Sic1 band after treatment with Cyclin B/Cdk1 (+) compared to control (−) (see Supplementary Material and Methods and Supplementary Figure 2).

## 3. Sic1 may inhibit Hxk2 activity *in vitro*

**FIGURE 2:**
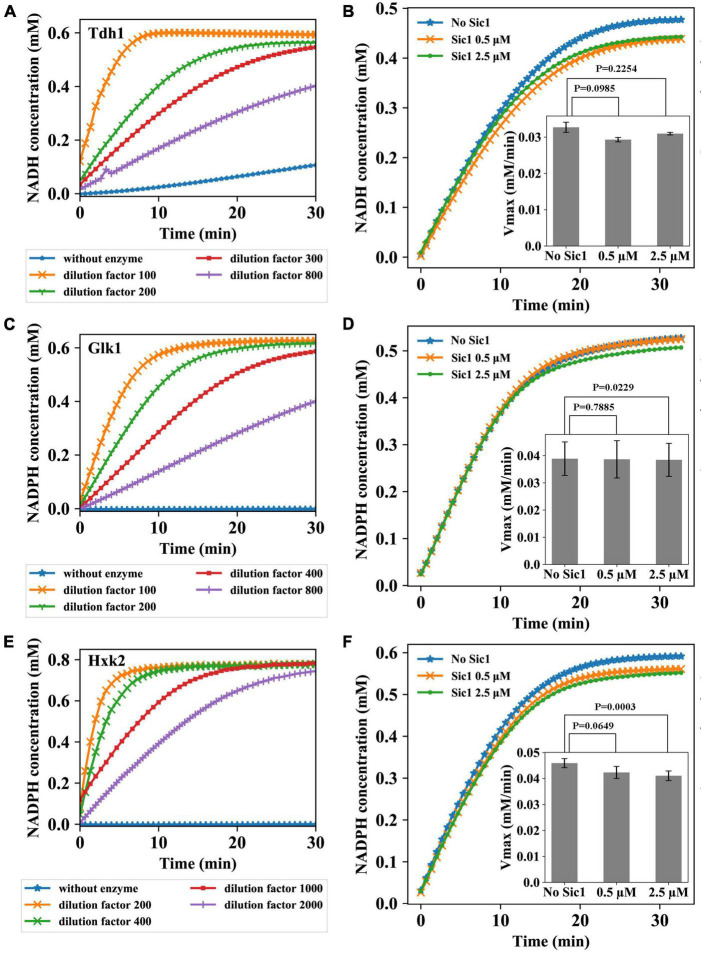
Sic1 effect on the glycolytic enzymes Tdh1, Glk1, and Hxk2. Enzymatic activity at different dilutions of Tdh1 **(A)**, Glk1 **(C)**, and Hxk2 **(E)** over time. Four different dilutions (Tdh1: 100–, 200–, 300–, 800–fold; Glk1: 100–, 200–, 400–, 800–fold; Hxk2: 200–, 400–, 1000–, 2000–fold) were used to determine the optimal enzyme concentrations. The activity of enzymes was measured under conditions optimized for maximal activity. Sic1 effect on Tdh1 **(B)**, Glk1 **(D)**, and Hxk2 **(F)**. The dilution factors of Tdh1, Glk1, and Hxk2 used in the experiments were 250, 400, and 2000, respectively. In panels **(B,D,F)**, the complete enzymatic reactions over time after adding Sic1 are reported, with the effect of Sic1 on the *V*_*max*_ of the enzymes shown as insets. For each enzyme, *V*_*max*_ was calculated using the curve obtained after the first 3 min of the reaction. The experiments were repeated in triplicate with two Sic1 conditions, 0.5 and 2.5 μM. Error bars on the histograms represent standard deviations from the mean of three independent experiments; *p*-values are indicated on the histograms.

In the second part of the study, we have explored the possibility for Sic1, the stoichiometric inhibitor of cyclin/Cdk1 complexes, to act directly on specific glycolytic enzymes. Specifically, the activity of three glycolytic enzymes, Tdh1, Glk1, and Hxk2, was measured under conditions optimized for maximal activity (see Supplementary Material and Methods). The activity of these enzymes was first tested at various enzymes dilutions, to identify the optimal conditions to carry the enzymatic assays. The choice of these dilutions was based on the protein concentration assay and the information retrieved in literature. Different dilutions were chosen because, for testing Sic1 effect on the enzymes, a definite enzyme concentration needs to be identified at which the reaction would neither occur too fast to prevent measurements, nor too slowly so that NADH or NADPH would be produced at a too low rate to enable measurements. The results are shown in Figure 2.

Four different dilutions of Tdh1 were used (Figure 2A). Contrarily to the chosen dilutions which showed time dependency, a control without adding any of the Tdh1 preparation did not show the expected time independence of the absorption at 340 nm (0.0 mM NADH during the complete experiment), which was instead observed in more experiments with Tdh1 (data not shown). This may have been due to contamination of other enzymes in the purified Tdh1 sample. From the experimental result, we chose to carry out the subsequent Sic1 assay using a Tdh1 dilution factor of 250. Also for Glk1 four different dilutions showed the expected time dependency (Figure 2C). As expected, the control curve, i.e., the one without enzyme, showed a persistent concentration of 0.0 mM NADPH, whereas the lowest dilution factor (100) resulted in the highest curve, i.e., the strongest reduction of NADPH. At this lowest dilution, the NADPH concentration virtually reached its plateau within 10 min. Conversely, the highest dilution (800–fold) resulted in the lowest curve, which did not reach the plateau during the time of the experiment. For the subsequent Sic1 assay, we chose to use a Glk1 dilution factor of 400, which approaches the plateau after about 30 min. Figure 2E reports the assay for Hxk2. The control reaction showed the expected concentration of 0.0 mM NADPH. The lowest dilution (200–fold) reached the plateau around 5 min after the start of the experiment, whereas the highest dilution (2000–fold) took the longest time to reach the plateau. For the subsequent Sic1 assay, we chose to use a Hxk2 dilution factor of 2000.

Prior to investigate the impact of Sic1 on the activity of the metabolic enzymes under investigation, Sic1 was purified (see Supplementary Material and Methods). The effect of Sic1 was then examined on Tdh1, Glk1, and Hxk2, through measuring the difference in the enzymatic activity before and after addition of Sic1 to the reaction mixture. It is expected that a decrease or an increase in the *V*_*max*_ activity of these metabolic enzymes would occur in case of an effect mediated by Sic1. When the enzyme is saturated with a substrate, the rate of the reaction is equal to *V*_*max*_. The enzymatic measurements were therefore conducted with substrate concentrations well above the *K_*m*_s* of each enzyme—under these conditions the enzyme is saturated, and the reaction rate is equal to *V*_*max*_. For example, *K*_*mGlucose*_ and *K*_*mATP*_ of Glk1 are equal to 0.028 and 0.05 mM, respectively (Laht et al., 2002), and the substrate concentrations used for measurements are 10 and 1 mM separately. This influence should be larger when a higher concentration of Sic1 would be added.

To test Sic1 effect on Tdh1 activity, a 250–fold dilution of the Tdh1 preparation was used. The result is shown in Figure 2B. A decrease in the *V*_*max*_ of Tdh1 is observed upon adding Sic1 to the enzymatic reaction, indicating that Sic1 may have a negative effect on the Tdh1 activity. However, this effect is relatively small—and has an inverse dependence on the concentration of Sic1 –, i.e., 8.5% reduction in rate with 0.5 μM Sic1, and about 5% reduction in rate with 2.5 μM Sic1. The result of testing Sic1 effect on Glk1 activity is shown in Figure 2D. No obvious difference in the activity of Glk1 can be observed after adding Sic1, at any used concentration, to the enzymatic reaction. Finally, Figure 2E shows the result of Sic1 effect on Hxk2 activity. The results for Hxk2 are somewhat similar, although with a slightly larger effect, to those observed for Tdh1. The two different concentrations of Sic1 tested decreased the Hxk2 enzymatic activity, which appears to be Sic1-dependent. In fact, an 8% reduction in rate is observed with 0.5 μM Sic1, whereas a 11% reduction in rate is observed with 2.5 μM Sic1.

In testing Sic1 effect on the yeast metabolic enzymes, two concentrations were used, i.e., 0.5 and 2.5 μM. Deshaies and Ferrell (2001) have indicated that the normal concentration of Sic1 in budding yeast cells may be around 1 μM; thus, additional concentrations of Sic1 within the range used might be tested in future experiments.

## 4. Discussion and perspectives

In this perspective, we shed light on the complex interconnections between cell cycle and metabolism, presenting initial investigation about some among these possible mechanisms that are mostly to be uncovered yet. Recent evidence in budding yeast pointed out that central carbon metabolism products synchronize their abundance profiles with cell division and reach their peak levels in definite cell cycle phases (Campbell et al., 2020; Takhaveev et al., 2023). Furthermore, it has been shown that cell cycle proteins directly modulate the activity of metabolic enzymes. Indeed, Cdk1 phosphorylation improves the activities of Gph1 (Zhao et al., 2016), Nth1 (Ewald et al., 2016; Zhao et al., 2016), Tgl4 (Ubersax et al., 2003; Kurat et al., 2009), and Pah1 (Santos-Rosa et al., 2005). In this study, we aimed to investigate mechanisms coordinating the cell cycle core machinery with central carbon metabolism in budding yeast. We reveal possible new routes through which cell cycle may regulate cellular metabolism that shall be further explored.

Specifically, we investigated the effect of some of the critical molecules governing the cell cycle machinery in budding yeast, i.e., the mitotic cyclin/Cdk1 complex and its stoichiometric inhibitor, on relevant glycolytic enzymes involved in cell division. First, *in vitro* phosphorylation assays using the mammalian Cyclin B/Cdk1 complex—functional homolog of the budding yeast Clb2/Cdk1—revealed the possible Cdk1-mediated phosphorylation of Fba1 and Pgk1, in addition to the known effect on Gph1 (used as control in the assays). We acknowledge these results have to be confirmed by further independent experiments. However, the distance between the higher and the main bands of Fba1 and Pgk1 are larger than those between the phosphorylated and non-phosphorylated Gph1 bands, suggesting that this higher bands represents hyper phosphorylation of Fba1 and Pgk1. In Figure 1, only a small fraction of Fba1 and Pgk1 displayed the migration shift while the majority of the enzyme exhibited a similar migration pattern as the control. A possible explanation for this observation is that, due to the purification step, the structure of the majority of the Fba1 or Pgk1 had been altered, so that only a small remaining fraction retaining the native structure could be phosphorylated. To derive a more definitive conclusion, the enzyme activity of Fba1 or Pgk1 may need to be assayed before and after coincubation with Cyclin B/Cdk1. In addition, mass spectrometry analysis to detect the presence of protein phosphates may be performed on the bands showing mobility shift (lanes 8, 20) as well as on the bands without mobility (lanes 7, 19), after isolating these from the SDS-PAGE gel. Peptide mapping of phosphorylated peptides, or the use of collision-induced dissociation (CID), electron transfer dissociation (ETD), or electron capture dissociation (ECD) (MS/MS or tandem MS) to detect the identity of protein phosphates on the glycolytic enzymes, will provide further details about the phosphorylation events. These further experiments may provide a better understanding about the phosphorylation status, thereby the potential functional implications, of Fba1 and Pgk1 in relation to the cyclin/Cdk activity.

Fba1 is likely to be a Cdk1 target. Indeed, Fba1 is an essential enzyme involved in both gluconeogenesis and glycolysis, and its mutations have been associated to slow growth and reduced competitive fitness. Phosphorylation of Fba1 by Cdk1 may potentially modulate its activity, thereby exerting control over the supply of metabolic intermediates, such as dihydroxyacetone phosphate (DHAP) and glyceraldehyde-3-phosphate (GAP). DHAP and GAP serve as substrates in various pathways, including the pentose phosphate pathway and lipid metabolism, that regulate critical events such as the formation of cellular membranes. Consequently, phosphorylation of Fba1 by Cdk1 could enable the cell to regulate the availability of these metabolites, when required, in different cell cycle phases. Further studies related to this finding may be conducted, such as for example testing the changes in the enzyme activity after phosphorylation by Cdk1, as well as *in vivo* experiment to examine the phosphorylation status of Fba1 in the different cell cycle phases.

In addition to Fba1, Pgk1 is another enzyme that potentially serves as substrate for Cdk1-mediated phosphorylation. Pgk1 is an essential enzyme involved in the glycolytic pathway, specifically catalyzing the conversion of 1,3-bisphosphoglycerate (BPG) to 3-phosphoglycerate (3PG) during glycolysis. This enzymatic reaction generates ATP through substrate-level phosphorylation, playing a crucial role in energy production. The phosphorylation of Pgk1 by Cdk1 may modulate its activity, impacting on glycolytic flux and ATP production. Therefore, changes in the activity of Pgk1 after phosphorylation may be further tested. Furthermore, Cdk1-mediated phosphorylation of Pgk1 may occur in specific cell cycle phases or under certain cellular conditions, thereby introducing an additional layer of regulation to the glycolytic pathway. Therefore, *in vivo* experiment to test the phosphorylation status of Pgk1 in the different cell cycle phases may add further knowledge in the regulation of critical steps in the central carbon metabolism.

We conducted *in vitro* enzymatic assays to test the regulatory effect of Cdk1 and Sic1 on metabolic enzymes. The results we obtained by the *in vitro* enzymatic assays, to investigate the effects of the cyclin/Cdk1 inhibitor Sic1 on metabolic enzymes, provide further insights into the potential connection between the cell cycle and metabolism. Specifically, the inhibition of the enzymatic activity of Hxk2 by Sic1 suggests an intriguing role for Sic1 in regulating the glycolytic pathway. Hxk2, an enzyme involved in the first step of glycolysis, catalyzes the phosphorylation of glucose to glucose-6-phosphate. The inhibition of Hxk2 by Sic1 implies a mechanism by which the cell cycle may modulate glucose utilization and energy production. We acknowledge this result is based on *in vitro* enzymatic assays, and further experiments are necessary to confirm and extend the observation in a physiologically relevant context.

Altogether, our findings suggest a role for Cdk1 in modulating Fba1 and Pgk1 enzyme activity, and for Sic1 in modulating Hxk2 enzymatic activity. Although these finding provide possible new links between cell cycle regulators and metabolism, the understanding of the mutual control between cell cycle regulators and metabolic molecules is still limited, as well as limited is the literature data available. Substantial efforts are needed to fully elucidate the intricacies of this complex, interconnected relationship. Investigating the regulatory mechanisms and functional consequences of the interaction between cell cycle and metabolism will contribute to the understanding of their intimate coordination that regulate cellular vital functions.

## Data availability statement

The original contributions presented in the study are included in the article/Supplementary material, further inquiries can be directed to the corresponding author/s.

## Author contributions

MB: Conceptualization, Formal analysis, Funding acquisition, Investigation, Methodology, Project administration, Supervision, Visualization, Writing – original draft. YZ: Formal analysis, Investigation, Methodology, Visualization, Writing – original draft.
