# Supplementary material for: Exploring cell cycle-mediated regulations of glycolysis in budding yeast
Source: Front Microbiol. 2023 Oct 11;14:1270487. doi: 10.3389/fmicb.2023.1270487 (PMC10598772; doi:10.3389/fmicb.2023.1270487)
Supplement: Supplementary file 1 [file Data_Sheet_1.pdf]

Supplementary Material for

Exploring cell cycle-mediated regulations of glycolysis in budding yeast

Yanfei Zhang[1,2], Matteo Barberis[1,2,3*]

[1]Molecular Systems Biology, School of Biosciences, Faculty of Health and Medical Sciences, University of Surrey, Guildford, Surrey GU2 7XH, UK

[2]Synthetic Systems Biology and Nuclear Organization, Swammerdam Institute for Life Sciences, University of Amsterdam, Amsterdam 1098 XH, The Netherlands

[3]Centre for Mathematical and Computational Biology, CMCB, University of Surrey, Guildford, Surrey GU2 7XH, UK

*** Correspondence:**
Matteo Barberis
m.barberis@surrey.ac.uk ; matteo@barberislab.com

Supplementary Materials and Methods

Strain construction and growth conditions

Yeast strains BY4741 (*MATa his3Δ1 leu2Δ0met15Δ0ura3Δ0*) were grown aerobically in YPD medium at 30 °C, shaking at 180 rpm overnight. The genomic DNA was extracted and used as the template for amplification of the Tdh1, Glk1 and Hxk2, Fba1, and Gph1 of *S. cerevisiae*. pET6xHN-based plasmids were constructed for the purification of the enzymes with histidine residues added to their N-terminus (**Supplementary Table 1**). The genes were amplified using the oligonucleotides shown in **Supplementary Table 2**. After amplification, the genes were inserted into pET6xHN vectors using the Gibson assembly method. All *E. coli* strains were routinely grown using either Luria-Bertani (LB) liquid broth or solid LB with agar at 18 °C or 37 °C, respectively. The LB media was supplemented with ampicillin at 0.1 mg/mL for maintaining pET6xHN derived plasmids.

Protein expression and purification

To isolate the metabolic enzymes, bacteria containing the corresponding pET6xHN plasmids were cultured in liquid medium. After the induction of protein expression in the bacterial cultures with IPTG, the proteins were purified as described in the following.

To purify the yeast enzymes, the corresponding plasmids were transformed into *E. coli* BL21 (DE3). Single colonies were inoculated into LB medium with 0.1 mg/mL of ampicillin and grown at 37 °C until the OD_{600} of the culture reached 0.6–0.8. Cultures were cooled to 18 °C, and IPTG was added to a final concentration of 1 mM. After further growth at 16 °C for 14–16 h, the cells were harvested by centrifugation at 6,000 g for 30 min at 4 °C. The supernatant was discarded. The pellet was suspended in buffer A (150 mM Tris–HCl, 150 mM NaCl and 20 mM imidazole, 1 mM DTT, pH 7.5), and the cells were disrupted by sonication for 6 min with 10 seconds of bursts with 10 seconds of rest on ice in between. Then the cell lysate was centrifuged at 12,000 g for 30 min at 4 °C to remove cell debris. The purification process was conducted on Ni–NTA gravity columns (Thermo Fisher Scientific). After the supernatant had been loaded onto the column, the target protein was eluted with the buffers in the following sequence: 3 times 3 column volumes of buffer A, and then elution buffer with different imidazole concentrations. The elution fractions were selected for SDS-PAGE. The fraction with pure protein was subjected to dialysis to remove imidazole. After purification, the purified enzymes were quantitated in terms of protein concentration by the Bradford method using BSA as reference (Bradford, 1976). Their purity was assessed by SDS-PAGE. The purification was conducted by affinity chromatography, making use of the His-tag fused to protein as specified by the metabolic genes, and the SDS-PAGE of these enzymes is shown in **Supplementary Figure 1A**. The complete purity of a protein is indicated by the presence of one single band visible per sample. Indeed, in **Supplementary Figure 1A**, a single band is recognizable for the His-tagged Glk1, Hxk2, Fba1 and Tdh1, indicating that these fractions are pure. This holds also for the His-tagged Pfk27 and Gph1, where the main band accounts for the majority of the proteins, although some lighter bands are also visible. The His-tagged metabolic enzymes Zwf1, Pgi1 and Pgk1 of *S. cerevisiae* were purchased from Sigma-Aldrich, and the enzymatic assays were conducted similarly to those of the in-house purified enzymes. The SDS-PAGE of the purchased enzymes shown in **Supplementary Figure 1B** indicates that the main band visible in each fraction accounts for the majority of the proteins, although some lighter bands are also detected.

Sic1 was purified tagged to GST. For this reason, the Sic1 purification process was similar to that described above for the purification of the metabolic enzymes, except for three differences: (i) PBS

buffer was used instead of buffer A to suspend the cell pellet; (ii) Glutathione Agarose Resin (Thermo Fisher Scientific) was used instead of Ni-NTA resin; and (iii) in the elution step, the elution buffer used was PBS at different concentrations of reduced glutathione. The purification of Sic1 was conducted by affinity chromatography, making use of the GST-tag fused to the *S. cerevisiae SIC1* gene, and the SDS-PAGE is shown in **Supplementary Figure 1B**. The band expected for Sic1 accounts for the majority of the protein, although a few lighter bands are also detected. The lighter bands correspond to the degradation of Sic1 and/or to Sic1 without GST, as Sic1 is known to be a relatively unstable protein. The upper band appears to be characteristic in Sic1 purification.

In vitro phosphorylation assay and Phos-tag SDS-PAGE

Around 5 µg of metabolic enzyme were mixed with 80 ng of Cyclin B/Cdk1 (Sigma-Aldrich) in 1× Reaction buffer (40 mM MOPS/NaOH pH7.0, 1mM EDTA) supplemented with 0.5 mM ATP and incubated at 30 °C for about 1 h. The reaction was ended by adding loading buffer (ThermoFisher Scientific) and heating for 5 min in 95 °C. The phosphorylated proteins were resolved in 8% SDS-PAGE gel with 100 µM of acrylamide-pendant Phos-tagTM ligand (Wako). In phosphate affinity SDS-PAGE phos-tag Acrylamide and manganese chloride are incorporated into SDS-PAGE resolving gel, and the phos-tag then binds the phosphate groups of proteins in the sample. The bound phos-tag decreases the migration speed of the phosphorylated proteins, enabling separation of phosphorylated and non-phosphorylated forms.

Enzymatic assays

The activity of three glycolytic enzymes, Tdh1, Glk1, and Hxk2, was measured under conditions optimized for maximal activity (Van Hoek, et al., 1998; Van Eunen et al., 2010).

The activity of Tdh1 was measured at 30 °C in a coupled assay with phosphoglycerate kinase (Pgk1) by following the NADH production spectroscopically at 340 nm. The reaction mixture for 200 µL of the assay buffer contained 2 mM GAP, 2 mM NAD^+, 10 mM Na_2HPO_4, 1 mM ADP, 1.5 mM $MgSO_4$, 1 mM EDTA, 22.5 U/mL Pgk1, 1 mM DTT, 50 mM Tris-HCl, pH 7.5, and an appropriate amount of Tdh1. 190 µL of the reaction mixture were added to a 96-wells plate (microplate 96-wells, F- bottom, Greiner). The plate was out into the plate reader (Multiskan go, Thermo scientific) and warmed to 30 °C for approximately 2 min. Then 10 µL of the Tdh1 suspension (at different dilutions) were added to trigger the reaction. After short mixing, the plate was read in a kinetic loop at 340 nm with 100 runs in approximately half an hour. In the meantime, a standard curve of NADH was prepared. To find the optimal condition for testing Sic1's effect on enzyme activity, different dilutions (100–, 200–, 300–, and 800–fold) were prepared from the Tdh1 stock suspension (at a protein concentration of 13 mg/mL). The Tdh1 stock suspension it was diluted into aqueous 50 mM of Tris-HCl at pH 7.5. A reaction adding buffer instead of the enzyme was used as a control. The activities of Glk1 and Hxk2 were measured in a coupled assay with Glucose-6-phosphate dehydrogenase by following the NADPH production at 340 nm at 30 °C. The reaction mixture for 200 µL of the assay buffer included 10 mM Glucose, 1 mM ATP, 1 mM $NADP^+$, 5 mM $MgCl_2$, 1.8 U/ml Glucose-6-phosphate dehydrogenase, 1mM DTT, 100 mM Tris-imidazole buffer, pH 7.5, and an appropriate amount of enzyme. The subsequent steps were the same as in the Tdh1 activity assay, with the only difference being the Glk1 and Hxk2 dilutions. For Glk1 (stock concentration of 17 mg/mL) the dilutions were 100–, 200–, 400–, and 800–fold. For Hxk2 (stock concentration of 15 mg/mL) the dilutions were 400–, 500–, 1000–, and 2000–fold.

For testing Sic1's effect on the aforementioned enzymes, Sic1 was added into the reaction mixture before adding the enzymes.

Supplementary Tables

Supplementary Table 1. Strains and plasmids used in this study.

Strains and plasmids	Description	Source
BY4741	The genome of this strain was used as the template for amplification of genes.	Euroscarf
E. coli DH5a	This strain was used for the clone of the plasmid.	NEB
E. coli BL21	This strain was used for enzyme protein expression.	NEB
pET6xHN	This plasmid was used for construction of other plasmids.	NEB
pET6xHN-*glk1*	N-terminal His-tagged *glk1*, inserted between Not1 and SalI sites of pET6xHN	This study
pET6xHN-*hxk2*	N-terminal His-tagged *hxk2*, inserted between Not1 and SalI sites of pET6xHN	This study
pET6xHN-*fba1*	N-terminal His-tagged *fba*1 inserted between Not1 and SalI sites of pET6xHN	This study
pET6xHN-*pfk27*	N-terminal His-tagged *pfk27*, inserted between Not1 and SalI sites of pET6xHN	This study
pET6xHN-*tdh1*	N-terminal His-tagged *tdh1*, inserted between Not1 and SalI sites of pET6xHN	This study
pET6xHN-*gph1*	N-terminal His-tagged *gph1*, inserted between Not1 and SalI sites of pET6xHN	This study
pGEX6p2-*sic1*	N-terminal GST-tagged *sic1*, inserted between Not1 and SalI sites of pGEX6p2	Barberis laboratory

Supplementary Table 2. Oligonucleotides used in this study.

Enzyme	Primer	Sequence
Glk1	*glk1*_F1	5'- ATGATAAGGCCTCTGTCGACACGCGTCGACAATGTCATTCGACGAC-3'
	*glk1*_R1	5'-AGATTAATTAATTAAGCGGCCGC AAGGAAAAAAGCGGCCGCTTATAA-3'
Hxk2	*hxk1*_F1	5'- ATGATAAGGCCTCTGTCGACACGCGTCGACATGGTTCATTTAGGTC-3'
	*hxk*_R1	5'-AGATTAATTAATTAAGCGGCCGCAAGGAAAAAAGCGGGCCGCTTA-3'
Fba1	*Fba1*_F1	5'-ATGATAAGGCCTCTGTCGACGGTGTTGAACAAATCTTAAAG-3'
	*Fba1*_R1	5'-AGATTAATTAATTAAGCGGCCGCTTATAAAGTGTTAGTGGT-3'
Tdh1	*tdh1*_F1	5'- ATGATAAGGCCTCTGTCGACACGCGTCGACATGATCAGAATTGCTAT-3'
	*tdh1*_R1	5'-AAGGAAAAAAGCGGCCGCTTATAAAGTGTTAGTGGTAC-3'
Pfk27	*pfk27*_F1	5'-ATGATAAGGCCTCTGTCGACGGTGGTTCTTCCGATTCAGA-3'
	*pfk27*_R1	5'-AGATTAATTAATTAAGCGGCCGCTCAAGCAAATCCGTTGCT-3'
Gph1	*gph1*_F1	5'-ATGATAAGGCCTCTGTCGACCCGCCAGCTAGTACTAGTACT-3'
	*gph1*_R1	5'-AGATTAATTAATTAAGCGGCCGCCTAAGTCACTGGTTCAACGT-3'
pET6xHN	*pET6xHN*_F1	5'-GCGGCCGCTTAATTAATTA-3'
	pET6xHN_R1	5'-GTCGACAGAGGCCTTATCAT-3'

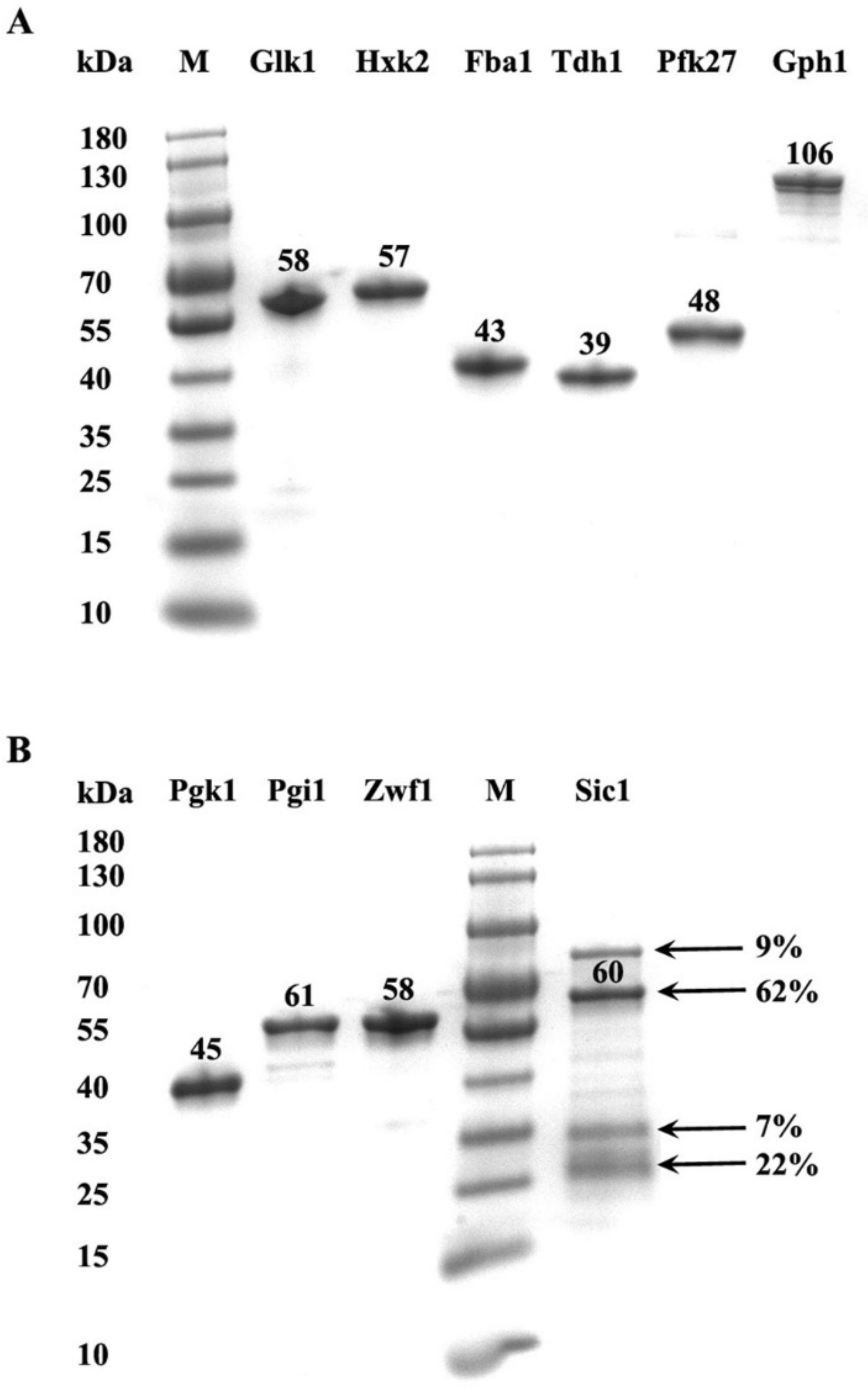

Supplementary Figure 1. SDS-PAGE of the proteins. **(A)** SDS-PAGE of the His-tag-purified Glk1, Hxk2, Fba1, Tdh1, Pfk27, and Gph1. **(B)** SDS-PAGE of the purified GST-Sic1 and of the proteins Zwf1, Pgi1, and Pgk1 purchased from Sigma-Aldrich. Molecular weights are indicated for each purification band. For Sic1, the % of the various bands is indicated (software ImageJ was used to provide quantitative values of each band for the percentage contribution to the total intensity).

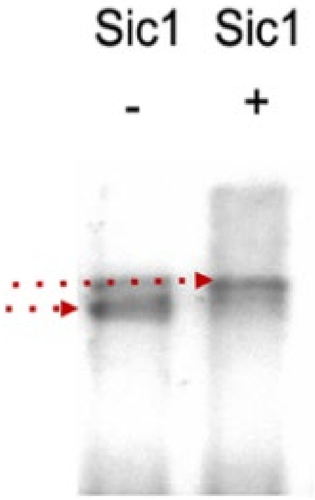

Supplementary Figure 2. Cyclin B/Cdk1 phosphorylation assay of Sic1 purified from *E. coli* and treated with human Cyclin B/Cdk1 *in vitro*, then analyzed on Phos-tag SDS-PAGE gels. (+) and (–) indicate the presence and absence of Cyclin B/Cdk1 in the assay, respectively. Mn^{2+}– Phos-tag^{TM} preferentially captures phospho-monoester dianions ($-OPO_3^{2-}$) bound to proteins, resulting in a mobility downshift of the phosphorylated proteins on the gel.
